# Dose-response association of leisure time physical activity with mortality in adults with major chronic diseases

**DOI:** 10.3389/fnut.2022.1048238

**Published:** 2022-12-21

**Authors:** Jiahong Sun, Han Wu, Min Zhao, Costan G. Magnussen, Bo Xi

**Affiliations:** ^1^Department of Epidemiology, School of Public Health, Cheeloo College of Medicine, Qilu Hospital, Shandong University, Jinan, Shandong, China; ^2^Department of Nutrition and Food Hygiene, School of Public Health, Cheeloo College of Medicine, Shandong University, Jinan, Shandong, China; ^3^Baker Heart and Diabetes Institute, Melbourne, VIC, Australia; ^4^Research Centre of Applied and Preventive Cardiovascular Medicine, University of Turku, Turku, Finland; ^5^Centre for Population Health Research, University of Turku, Turku University Hospital, Turku, Finland

**Keywords:** physical activity, mortality, dose-response, chronic diseases, epidemic

## Abstract

We aimed to evaluate the association between leisure-time physical activity (PA) and mortality risk in adults with major chronic diseases. A total of 170,579 adults with major chronic diseases aged 30–84 years from the U.S. National Health Interview Surveys (1997–2014) with linkage to the National Death Index (NDI) through December 31, 2015 were included in this study. During a median follow-up of 7.25 years, 36,914 adults with chronic diseases died from all causes, 8,767 died from cardiovascular disease (CVD), and 9,090 died from cancer. Compared with participants with no leisure-time PA, those with a low level (10–59 min/week) of total leisure-time PA had a 23% [hazard ratio (HR) 0.77, 95% confidence interval (CI) 0.73–0.82] reduced risk of all-cause mortality. Adults with higher levels of leisure time had more reduced risk of all-cause mortality, as well as CVD-specific and cancer-specific mortality. Adults with leisure-time PA ≥ 1,500 min/week had more reduced risk of CVD-specific mortality (61%) but less reduced risk of cancer-specific mortality (29%) compared with the reduced risk of all-cause mortality (43%). There was an inversely non-linear dose-response relationship between leisure-time PA and all-cause and cause-specific mortality. Reduced risk of all-cause and cancer-specific mortality between leisure-time light-to-moderate PA and vigorous-intensity PA time were largely comparable. Low and high levels of leisure-time PA showed substantial survival benefits compared with no leisure-time PA in adults with major chronic diseases. The light-to-moderate-intensity leisure-time PA is largely comparable with vigorous PA to provide survival benefits for all-cause and cancer-specific mortality.

## Introduction

Leisure-time Physical activity (PA) has a significant health benefit for individuals of all ages. It has been estimated that physical inactivity accounts for approximately 10% of premature death or more than 5.3 million deaths worldwide in 2008 (1). In the United States (U.S.), inadequate levels of PA were associated with 8.7% of total health care expenditure (2). The 2018 PA Guidelines for Americans (3), in accordance with the 2008 PA Guidelines (4) and the 2020 World Health Organization PA guidelines (5), recommend a minimum of 150 min of moderate-intensity PA (e.g., bicycling or dancing) or 75 min of vigorous-intensity PA (e.g., fast running or swimming) per week or an equivalent combination. These guidelines are applicable for all healthy adults and those with chronic non-communicable diseases [e.g., cardiovascular disease (CVD), diabetes, or cancer]. However, in 2018, only half of U.S. adults (54.2%) met the recommendations of the PA Guidelines (6).

Systematic reviews and meta-analyses have provided strong evidence that both low and high amounts of PA could reduce the risk of mortality in generally healthy adults (7–11). For example, a systematic review and meta-analysis that included 9 cohort studies and 122,417 adults aged 60 years or older found that participants who engaged in low-level [1–499 metabolic equivalents (METs)] -minutes/week, one MET is equivalent to the energy expenditure or the resting metabolic rate when sitting quietly and being awake. Moderate- and vigorous-intensity activities have MET values of 3∼5.9 METs and 6 or greater METs, respectively (12) and high-level (more than 1,000-MET minutes/week) moderate-to-vigorous-intensity leisure-time PA had 22% and 35% reduced risk of all-cause mortality, compared with those classified as inactive (8). In addition, a prospective study among 88,140 healthy U.S. adults suggested the benefits of leisure-time PA at any dose on all-cause, CVD-, and cancer-specific mortality, irrespective of PA intensity (13). Recently, we found an 11%, 29%, and 40% reduction in all-cause mortality in general U.S. adults who met the recommended 2018 muscle-strengthening activity, aerobic activity, and both, respectively, compared to those who did not meet the recommendation (14).

The achieved benefits from leisure-time PA among individuals with some chronic disease (e.g., CVD) seemed to be stronger than that among those without any chronic conditions (15). However, there is limited data on the dose-response association between leisure-time PA time and all-cause and cause-specific mortality in adults with chronic diseases, with inconsistent results (16–19). For instance, one study with a median follow-up of 3.7 years including 15,486 adults with stable coronary heart disease from 39 countries reported an inverse dose-response association between leisure-time PA levels and all-cause and CVD-specific mortality in adults with stable coronary heart disease (18), whereas another study found evidence of increased cardiovascular mortality in coronary heart disease adults who engaged in daily strenuous PA, compared with those who engaged in low levels of PA of 2–4 times/week (19). Inconsistent findings of the association between PA and cancer-specific mortality were also found among adults with cancer (16, 17). Overall, it remains unclear how different doses and intensities of PA time impact mortality risk in adults with chronic diseases. In addition, most previous studies and reviews were performed in adults with a specific chronic condition without consideration of leisure-time PA effects among those with chronic conditions on mortality risk. Therefore, it may be useful to examine the effect of leisure-time PA on mortality risk in adults with chronic diseases.

In this study, we examined the dose-response association between leisure-time PA and all-cause and cause-specific mortality among U.S. adults with major chronic diseases.

## Materials and methods

### Study population

The National Health Interviews Survey (NHIS) is an annual national cross-sectional household survey of the health status of a civilian and non-institutionalized U.S. population, conducted by the National Center for Health Statistics since 1957. A complex stratified and multistage sampling design was used to acquire information on demographics, health, and lifestyle behaviors among the sample participants through personal household interviews. More details on NHIS, including methodology, weighting, informed consent procedures, and the public availability of data can be found online.^1^ The NHIS data are de-identified and do not include any protected health information, and the available data are public and exempt under the ethical board review of the corresponding author’s institution.

According to the question of “Have you ever been told by a doctor or other health professional that you have diabetes?” as well as the same questions on hypertension, heart disease, stroke, and cancer, a total of 185,064 participants aged 30–84 years with self-reported chronic diseases were included from the NHIS during 1997 and 2014 linked to the National Death Index (NDI) (20) up to December 31, 2015. Due to the major revision of questionnaires in 1997, we used the available NHIS data starting from 1997 to maintain a comparison between all surveys. Among the 185,064 participants, 14,485 were excluded because of being pregnant (*n* = 425), or were missing data on leisure-time PA (*n* = 5,279) or potential covariates (*n* = 8,781, i.e., demographic variables and lifestyle factors), leading to a final analytic sample of 170,579 adults with major chronic diseases.

### Exposures

All study participants were administered standard questionnaires that collected information on frequency (times/week) and duration (minutes/time) of leisure-time PA during the past year. Frequency of light-to-moderate [e.g., slow walking or bicycling, defined as under 6 METs (4)] and vigorous-intensity [e.g., faster cycling and running, defined as more than 6 METs (4)] leisure-time PA that lasted at least 10 min was evaluated using the following questions: (1) light-to-moderate PA: “How often do you do light or moderate leisure-time PA for at least 10 min that cause heavy sweating or large increases in breathing or heart rate?” including “How many times per day, per week, per month, or per year” and “how long do you do such activities each time” (2) vigorous PA: the three questions on vigorous leisure-time PA were similar to that of light to moderate leisure-time PA. The questionnaire is available on the website of https://www.cdc.gov/nchs/nhis/data-questionnaires-documentation.htm. Total minutes/week of leisure-time PA was calculated by summing light-to-moderate and vigorous-intensity leisure-time PA [1 min of vigorous-intensity PA was equivalent to 2 min of moderate-intensity PA according to the PA Guidelines (4), e.g., 60 min of vigorous-intensity PA per week is similar to 120 min of moderate-intensity PA per week], which considered both the frequency and duration of each PA.

Study participants were categorized into the following eight groups according to the levels of leisure-time PA: 0 (totally sedentary), 10–59, 60–149, 150–299, 300–449, 450–799, 800–1,499, and ≥ 1,500 min/week, in accordance with previous publications (10, 11, 13). Moreover, leisure-time PA was further classified according to intensity (light-to-moderate and vigorous) as the following six groups: 0, 10–59, 60–149, 150–299, 300–599, and ≥ 600 min/week. Also, muscle-strengthening activity was defined according to self-reported response to the following question “How often do you do physical activities specifically designed to strengthen your muscles, such as lifting weights or doing calisthenics?”

### Outcomes

Data from the NHIS between 1997 and 2014 are linked to the mortality records in NDI (20) up to December 31, 2015. The all-cause and cause-specific mortality statuses were certified using a probabilistic matching algorithm, which yields a near-perfect agreement (98.5%) (21). Participants not matched to mortality records in the NDI were classified as being alive. The *International Classification of Disease-10th Revision* codes were used to define mortality, including all-cause mortality, CVD-specific mortality (codes I00 to I09, I11, I13, I20 to I51, and I60 to I69), and cancer-specific mortality (codes C00 to C97).

### Confounding variables

Several potential covariates were available from baseline questionnaires, including demographics, lifestyle factors, and disease status. Demographic variables included age, sex, race/ethnicity (non-Hispanic black, non-Hispanic white, Hispanic, or other), education level (less than high school, high school, or beyond high school), and marital status (married, widowed/divorced/separated, or never married). Lifestyle factors included weight status defined by body mass index [weight/(height^2^) (kg/m^2^), categorized as underweight, normal weight or overweight/obesity], drinking status (lifetime abstainer, former, light to moderate, or heavy drinking), and smoking status (never, former, or current smoking). The disease status included the number of chronic diseases (classified as 1, 2, 3, or more).

### Statistical analysis

Categorical variables were presented as percentages (%) and differences between groups were compared using the chi-square test. Multivariate Cox proportional-hazard regression models were used to calculate the hazard ratios (HRs) and 95% confidence interval (CIs) of leisure-time PA levels with all-cause and cause-specific mortality. In addition, associations were also evaluated and classified by the intensity of leisure-time PA. Three models that successively adjusted for potential confounding factors were considered. Model 1 adjusted for age, sex, and race/ethnicity; Model 2 adjusted for variables in Model 1 and additionally adjusted for education level and marital status. Model 3 adjusted for variables in Model 2 and additionally adjusted for body mass index, smoking status, drinking status, the number of chronic diseases, and muscle-strengthening activity a type of anaerobic exercise that increases skeletal muscle power, strength and mass, which is different from aerobic activity [i.e., leisure time PA (5)]. To quantitatively assess the dose-response association between leisure-time PA (as a continuous variable for analysis) and all-cause and cause-specific mortality, Cox regression models with restricted cubic splines (22) were performed with three knots at the 5th, 50th, and 95th percentiles of leisure-time PA. Additionally, subgroup analyses stratified by age, sex, and race/ethnicity between total leisure-time PA and all-cause mortality were conducted. To assess the stability of the results, two sensitivity analyses were performed. First, a sensitivity analysis was performed to assess the association between total leisure-time PA and all-cause mortality stratified by the specific chronic disease or the number of chronic diseases at baseline. Second, a sensitivity analysis was conducted to evaluate the association between total leisure-time PA and all-cause and cause-specific mortality by excluding those who died within the first 2 years. All data analyses were performed with SAS 9.3 (SAS Institute, Inc., Cary, North Carolina) and R version 3.3.3 (R Foundation for Statistical Computing, Vienna, Austria). A two-side *P*-value < 0.05 was indicated as a significant difference.

## Results

### Characteristics of study participants

The characteristics of 170,579 participants aged 30–84 years according to total leisure-time PA level are shown in Table 1. A significant difference was found for each descriptive characteristic across the eight leisure-time PA levels (all *P* < 0.0001). Compared with adults with chronic diseases who had no leisure-time PA, those who engaged in leisure-time PA at higher levels (from 10 to 59 min/week to ≥ 1,500 min/week) were more likely to be young, men, white, educated more than high school, married, normal-weight, or overweight, never or former smokers, light-to-moderate drinkers, and have less chronic diseases.

**TABLE 1:** Baseline characteristics according to total leisure-time physical activity level, NHIS 1997–2014.

	Leisure-time physical activity level, minutes/week								
	**0**	**10–59**	**60–149**	**150–299**	**300–449**	**450–799**	**800–1,499**	**≥ 1,500**	***P*-value**
***N***	78,629	8,870	23,587	20,862	13,904	12,641	8,065	4,021	
Sex,%									<0.0001
Men	45.2	42.3	44.6	48.6	52.0	58.1	61.4	65.7	
Women	54.8	57.7	55.4	51.4	48.0	41.9	38.6	34.3	
Age, years, %									<0.0001
30–49	24.5	30.2	29.7	31.2	33.0	36.8	33.8	33.7	
50–69	47.2	49.6	48.2	48.5	47.5	48.1	48.8	49.3	
70–84	28.3	20.2	22.1	20.3	19.5	15.0	17.4	16.9	
Race/ethnicity, %									<0.0001
White	70.9	75.8	77.0	78.7	78.8	80.4	78.1	79.5	
Black	15.3	12.8	11.4	9.9	9.6	9.6	9.9	9.8	
Hispanic	10.4	7.3	7.6	7.2	7.3	6.6	7.7	7.3	
Other	3.4	4.1	4.0	4.2	4.3	3.5	4.3	3.4	
Marital status, %									<0.0001
Married	57.9	62.5	64.4	67.0	67.2	69.5	68.2	66.7	
Divorced/separated /widowed	30.4	25.3	24.4	21.9	21.3	18.5	19.8	20.3	
Never married	11.7	12.2	11.2	11.1	11.5	12.0	12.0	12.9	
Education, %									<0.0001
<High school	27.7	15.6	13.8	11.4	10.5	7.8	10.7	12.4	
High school	35.1	31.0	30.1	26.9	25.1	22.5	25.7	28.2	
> High school	37.1	53.5	56.1	61.7	64.4	69.7	63.7	59.3	
Body mass index, kg/m^2^, %									<0.0001
<25.0	25.7	22.0	26.6	27.6	29.9	29.6	29.4	28.1	
25.0–29.9	33.7	35.1	36.0	39.2	39.1	40.4	41.3	40.9	
≥ 30.0	40.6	42.9	37.4	33.2	31.0	29.9	29.3	31.0	
Drinking, %									<0.0001
Lifetime abstainer	28.8	17.5	17.0	16.0	14.0	11.2	12.9	12.2	
Former drinker	26.7	22.9	20.9	18.3	18.0	15.0	17.2	17.7	
Light to moderate drinker	40.0	55.1	57.7	60.7	62.5	68.1	63.3	62.5	
Heavy drinker	4.6	4.5	4.5	5.0	5.5	5.6	6.5	7.6	
Smoking, %									<0.0001
Never	46.3	50.2	50.4	52.0	50.6	50.6	47.6	44.2	
Former	30.7	31.3	32.8	33.5	34.7	35.8	36.0	34.7	
Current	22.9	18.5	16.8	14.5	14.7	13.6	16.4	21.1	
No. of chronic diseases									<0.0001
1	55.5	61.4	63.3	65.8	67.7	70.5	68.7	69.8	
2	28.8	27.0	26.2	24.7	24.0	22.5	24.1	23.9	
≥ 3	15.7	11.6	10.6	9.5	8.2	7.0	7.2	6.4	

Data are presented as percentages (%).

### Associations between total leisure-time physical activity level and all-cause and cause-specific mortality

During a median follow-up of 7.25 years, there were 36,914 all-cause deaths, 8,767 CVD-specific deaths, and 9,090 cancer-specific deaths. The HRs with 95% CIs for all-cause and cause-specific deaths across the eight total leisure-time PA levels are shown in Table 2. After adjusting for all potential covariates, compared with adults with chronic diseases who had no leisure-time PA, those who engaged in less than the recommended level of leisure-time PA indicated in the PA Guidelines (i.e., < 150 min/week) had a reduced risk of all-cause mortality, with a 23% (HR 0.77, 95% CI: 0.73–0.82) reduction for 10–59 min/week of PA, and a 26% (HR 0.74, 95% CI: 0.71–0.77) reduction for 60–149 min/week of PA. Moreover, those who performed 1–2 times (150–299 min/week), 2–3 times (300–449 min/week), 3–5 times (450–799 min/week), 5–10 times (800–1,499 min/week) of the recommended PA level had progressively 36% (HR 0.64, 95% CI: 0.61–0.67), 40% (HR 0.60, 95% CI: 0.57–0.64), 42% (HR 0.58, 95% CI: 0.54–0.61), and 44% (HR 0.56, 95% CI: 0.52–0.60), respectively, reduced risk of all-cause mortality. Of note, those who performed ≥ 10 times the recommended leisure-time PA level (i.e., ≥ 1,500 min/week) still had 43% (HR 0.57, 95% CI: 0.52–0.63) reduced risk of all-cause mortality. Similar beneficial effects on CVD- and cancer-specific mortality were also observed across the different leisure-time PA levels less than 1,500 min/week. However, those who engaged in leisure-time PA ≥ 1,500 min/week had more reduced risk of CVD-specific mortality (HR 0.39, 95% CI: 0.31–0.50) but relatively less reduced risk of cancer-specific mortality (HR 0.71, 95% CI: 0.59–0.84), compared with the reduced risk of all-cause mortality. The association between total leisure-time PA level and all-cause mortality was similar to the summary data when stratified by age, sex, and race/ethnicity (Supplementary Table 1).

**TABLE 2:** Association between total leisure-time physical activity level and all-cause and cause-specific mortality.

	Leisure-time physical activity level, minutes/week							
	**0**	**10–59**	**60–149**	**150–299**	**300–449**	**450–799**	**800–1,499**	**≥ 1,500**
***N***	78,629	8,870	23,587	20,862	13,904	12,641	8,065	4,021
All-cause								
Deaths	22,739	1,560	4,308	3,287	2,009	1,459	1,043	509
Model 1	1.00	0.70 (0.66–0.75)	0.66 (0.64–0.69)	0.55 (0.53–0.57)	0.51 (0.49–0.54)	0.47 (0.44–0.50)	0.47 (0.44–0.51)	0.49 (0.44–0.54)
Model 2	1.00	0.73 (0.69–0.78)	0.70 (0.67–0.73)	0.59 (0.57–0.62)	0.56 (0.53–0.59)	0.52 (0.49–0.55)	0.51 (0.48–0.55)	0.52 (0.47–0.58)
Model 3	1.00	0.77 (0.73–0.82)	0.74 (0.71–0.77)	0.64 (0.61–0.67)	0.60 (0.57–0.64)	0.58 (0.54–0.61)	0.56 (0.52–0.60)	0.57 (0.52–0.63)
Cardiovascular disease								
Deaths	5,511	347	1,050	767	443	331	224	94
Model 1	1.00	0.62 (0.54–0.70)	0.63 (0.58–0.68)	0.48 (0.44–0.52)	0.44 (0.40–0.49)	0.41 (0.36–0.46)	0.39 (0.34–0.46)	0.31 (0.24–0.39)
Model 2	1.00	0.65 (0.57–0.74)	0.68 (0.63–0.74)	0.53 (0.49–0.59)	0.50 (0.45–0.56)	0.47 (0.41–0.53)	0.44 (0.38–0.52)	0.34 (0.27–0.43)
Model 3	1.00	0.69 (0.60–0.78)	0.74 (0.68–0.79)	0.58 (0.53–0.64)	0.55 (0.50–0.62)	0.54 (0.47–0.61)	0.50 (0.43–0.59)	0.39 (0.31–0.50)
Cancer								
Deaths	5,199	412	1,130	896	542	456	286	169
Model 1	1.00	0.73 (0.65–0.82)	0.69 (0.64–0.75)	0.58 (0.53–0.63)	0.54 (0.49–0.60)	0.55 (0.49–0.61)	0.48 (0.42–0.56)	0.62 (0.52–0.74)
Model 2	1.00	0.76 (0.68–0.86)	0.73 (0.68–0.79)	0.62 (0.57–0.67)	0.58 (0.52–0.65)	0.60 (0.54–0.67)	0.52 (0.45–0.60)	0.66 (0.56–0.79)
Model 3	1.00	0.80 (0.71–0.90)	0.77 (0.72–0.84)	0.67 (0.61–0.72)	0.63 (0.57–0.70)	0.66 (0.59–0.74)	0.56 (0.49–0.65)	0.71 (0.59–0.84)

Data are presented as hazard ratios (95% confidence intervals).

Model 1: Adjusted for sex, age, and race/ethnicity.

Model 2: Model 1 + education and marital status.

Model 3: Model 2 + body mass index, smoking, alcohol intake, number of chronic diseases, and muscle-strengthening activity.

### Sensitivity analysis of associations between total leisure-time physical activity level and all-cause and cause-specific mortality

Two sensitivity analyses were performed to test the stability of our findings. First, the association between total leisure-time PA and all-cause mortality stratified by type (i.e., hypertension, heart disease, stroke, diabetes, and cancer) or number of chronic diseases (i.e., 1, 2, ≥ 3 chronic diseases) at baseline yielded similar results (Supplementary Table 2). Second, the exclusion of adults with chronic diseases who died within the first 2 years had little effect on the risk of mortality from all-cause, CVD-, and cancer-specific outcomes (Supplementary Table 3).

### Dose-response relationship between leisure-time PA and all-cause and cause-specific mortality

**FIGURE 1:**
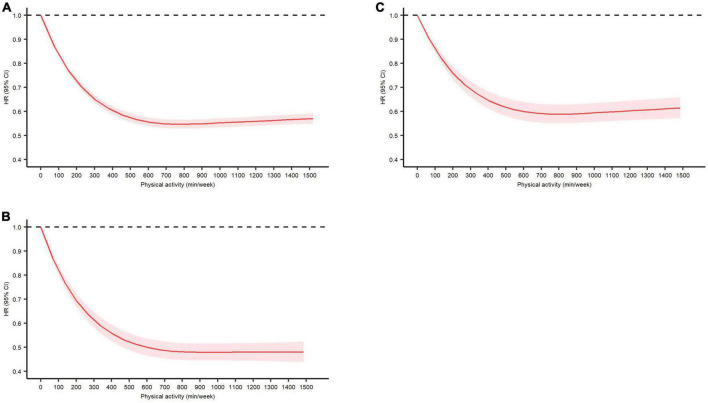
Dose-response relationship between total leisure-time physical activity (minutes/week) and **(A)** all-cause mortality, **(B)** cardiovascular disease-specific mortality, and **(C)** cancer-specific mortality in adults with major chronic diseases with adjustment for age, sex, and race/ethnicity, education level, marital status, body mass index, smoking status, drinking status, the number of chronic diseases, and muscle-strengthening activity. Data are presented as hazard ratio and 95% confidence interval.

The dose-response relationship between leisure-time PA and all-cause and cause-specific mortality (adjusting for all potential covariates) is presented in Figures 1A–C. An inversely non-linear dose-response relationship was found between leisure-time PA and all-cause mortality (*P* for the non-linear test < 0.0001, Figure 1A), CVD-specific mortality (*P* for the non-linear test < 0.0001, Figure 1B), and cancer-specific mortality (*P* for the non-linear test < 0.0001, Figure 1C). Compared with adults with chronic diseases who had no leisure-time PA, the beneficial effects of leisure-time PA on all-cause, CVD-, and cancer-specific mortality were found to start from a low dose, increased steeply up to 300 min/week, and slowly up to 600 min/week. The reduced risk of CVD-specific mortality remained stable from 600 min/week to ≥ 1,500 min/week, whereas the reduced risk of all-cause mortality and cancer-specific mortality recovered slightly from 600 min/week to ≥ 1,500 min/week (Figures 1A–C).

### Associations between leisure-time PA and all-cause and cause-specific mortality classified by two PA intensities

The association between leisure-time PA and all-cause and cause-specific mortality classified by two PA intensities is presented in Table 3. Low levels (10–59 min/week) of light-to-moderate and vigorous-intensity leisure-time PA could reduce the risk of all-cause mortality by 22% (HR 0.78, 95% CI: 0.74–0.82) and 24% (HR 0.76, 95% CI: 0.70–0.83), respectively. Increased levels of light-to-moderate or vigorous-intensity leisure-time PA further reduced the risk of all-cause mortality with the risk reduction observed for both intensities largely comparable. Those with the highest levels (≥ 600 min/week) of light-to-moderate or vigorous-intensity leisure-time PA tended to have a similar risk reduction (27%) in all-cause mortality. Similar patterns were found for cancer-specific mortality. However, the reduced risk for CVD-specific mortality among those with ≥ 600 min/week of vigorous-intensity leisure-time PA was greater than those with ≥ 600 min/week of light-to-moderate PA (54% vs. 32%).

**TABLE 3:** Association between leisure-time physical activity level and all-cause and cause-specific mortality by the intensity of physical activity.

	Leisure-time physical activity level, minutes/week					
	**0**	**10**–**59**	**60**–**149**	**150**–**299**	**300**–**599**	** ≥ 600**
Light-to-moderate						
***N***	89,585	13,101	32,338	18,723	11,001	5,831
All-cause						
Deaths	24,148	1,880	5,082	3,019	1,822	963
Model 1	1.00	0.71 (0.68–0.75)	0.71 (0.68–0.74)	0.63 (0.61–0.66)	0.66 (0.62–0.69)	0.67 (0.63–0.72)
Model 2	1.00	0.74 (0.70–0.78)	0.75 (0.72–0.77)	0.67 (0.64–0.70)	0.69 (0.65–0.73)	0.71 (0.66–0.76)
Model 3	1.00	0.78 (0.74–0.82)	0.78 (0.76–0.81)	0.70 (0.67–0.73)	0.72 (0.68–0.76)	0.73 (0.68–0.78)
Cardiovascular disease						
Deaths	5,821	408	1,222	701	5,821	408
Model 1	1.00	0.62 (0.55–0.70)	0.68 (0.64–0.73)	0.56 (0.51–0.61)	0.56 (0.50–0.63)	0.60 (0.51–0.69)
Model 2	1.00	0.65 (0.58–0.74)	0.74 (0.68–0.79)	0.61 (0.55–0.67)	0.61 (0.55–0.69)	0.64 (0.55–0.74)
Model 3	1.00	0.69 (0.62–0.78)	0.78 (0.73–0.84)	0.65 (0.59–0.71)	0.65 (0.58–0.73)	0.68 (0.59–0.79)
Cancer						
Deaths	5,606	526	1,353	837	499	269
Model 1	1.00	0.75 (0.68–0.84)	0.73 (0.68–0.78)	0.68 (0.63–0.74)	0.71 (0.63–0.79)	0.75 (0.65–0.86)
Model 2	1.00	0.78 (0.70–0.87)	0.77 (0.71–0.82)	0.72 (0.66–0.78)	0.74 (0.66–0.83)	0.78 (0.67–0.89)
Model 3	1.00	0.82 (0.73–0.91)	0.80 (0.75–0.86)	0.75 (0.69–0.81)	0.76 (0.68–0.85)	0.79 (0.68–0.90)
Vigorous						
***N***	125,648	6,404	17,199	11,717	6,577	3,034
All-cause						
Deaths	32,158	668	1,851	1,211	686	340
Model 1	1.00	0.69 (0.63–0.75)	0.62 (0.59–0.66)	0.56 (0.52–0.60)	0.56 (0.51–0.61)	0.66 (0.58–0.74)
Model 2	1.00	0.72 (0.66–0.78)	0.66 (0.63–0.70)	0.59 (0.56–0.64)	0.59 (0.54–0.65)	0.68 (0.60–0.77)
Model 3	1.00	0.76 (0.70–0.83)	0.71 (0.68–0.75)	0.64 (0.60–0.68)	0.65 (0.59–0.71)	0.73 (0.64–0.83)
Cardiovascular disease						
Deaths	7,757	144	401	272	136	57
Model 1	1.00	0.66 (0.55–0.81)	0.58 (0.52–0.65)	0.51 (0.44–0.58)	0.47 (0.39–0.57)	0.39 (0.29–0.53)
Model 2	1.00	0.70 (0.58–0.85)	0.63 (0.56–0.70)	0.56 (0.49–0.64)	0.51 (0.42–0.62)	0.41 (0.31–0.55)
Model 3	1.00	0.74 (0.61–0.90)	0.69 (0.61–0.77)	0.61 (0.53–0.70)	0.58 (0.48–0.70)	0.46 (0.34–0.62)
Cancer						
Deaths	7,673	178	564	341	216	118
Model 1	1.00	0.67 (0.57–0.79)	0.67 (0.61–0.74)	0.55 (0.49–0.63)	0.62 (0.53–0.74)	0.80 (0.64–1.00)
Model 2	1.00	0.69 (0.59–0.82)	0.70 (0.64–0.78)	0.59 (0.52–0.66)	0.65 (0.55–0.77)	0.82 (0.66–1.03)
Model 3	1.00	0.74 (0.62–0.87)	0.76 (0.68–0.84)	0.64 (0.56–0.72)	0.71 (0.60–0.84)	0.86 (0.68–1.07)

Data are presented as hazard ratios (95% confidence intervals).

Model 1: Adjusted for sex, age, and race/ethnicity.

Model 2: Model 1 + education and marital status.

Model 3: Model 2 + body mass index, smoking, alcohol intake, number of chronic diseases, and muscle-strengthening activity.

## Discussion

In this large prospective study including a nationally representative sample of 170,579 U.S. adults with major chronic diseases, we found that those who engaged in low level (even 10–59 min/week) or high level of leisure-time PA had a reduced risk of all-cause, CVD- and cancer-specific mortality compared with those who had no leisure-time PA. There was also an inversely non-linear association between PA dose and risk of all-cause, CVD- and cancer-specific mortality. The reduced risk of all-cause, CVD- and cancer-specific mortality increased steeply up to 300 min/week and slowly up to 600 min/week. From 600 min/week to ≥ 1,500 min/week, the reduced risk of CVD-specific mortality remained stable, whereas the reduced risk of all-cause mortality and cancer-specific mortality recovered slightly. In addition, we found that adults with ≥ 600 min/week vigorous-intensity PA could achieve more reduced risks of CVD-specific mortality than those with light-to-moderate PA, whereas the reduced risk was largely comparable for all-cause and cancer-specific mortality. Our findings have important public health and clinical implications as they suggest that individuals with chronic diseases who engage in leisure-time PA (even light-to-moderate-intensity) can have significant survival benefits.

Data from several prospective studies in the general population have suggested that adults with both low- and high- levels of PA had a reduced risk of mortality (7–11, 13). However, the association between leisure-time PA levels and mortality in adults with chronic diseases has been less investigated, and the results have been inconsistent (16, 18, 19, 23). Similar to our findings, a global cohort of 15,486 adults with stable coronary heart disease (a median follow-up of 3.7 years) has shown that each doubling volume of PA was associated with reduced risk of all-cause and CVD-specific mortality (0 as the reference, from 0 to 5 to > 160 MET-hours/week) (18). Among 1,038 Germans with stable coronary heart disease (over 10 years of follow-up), low-frequency PA (2–4 times/week) was associated with a reduced risk of CVD-specific mortality (19). However, among 1,117 Norwegian adults with atrial fibrillation (7–9 years of follow-up), those with insufficient PA levels < 150 min of moderate-intensity PA and < 75 min of vigorous-intensity PA per week only had a reduced risk of all-cause mortality but not of CVD-specific mortality (22).

In addition, a meta-analysis by Je et al. including 6 cohort studies (with 3.8–11.9 years of follow-up) showed that compared with adults with colorectal cancer who performed low levels of PA, only those with high PA levels had a reduced risk of cancer-specific mortality (16). In contrast, a meta-analysis of 35 cohort studies including 69,011 cancer survivors (with a median follow-up of 2.74–13 years) demonstrated even those who engaged in a minimum of 2.5 h/week of PA had a reduced risk of cancer mortality (17). We found that both low and high levels of leisure-time PA were inversely associated with cancer-specific mortality and the inconsistent findings of these studies mentioned above might be due to differences in recruitment, chronic diseases, and cancer types, basic characteristics of adults, statistical power, duration of follow-up, and adjustment of potential covariates.

We additionally found an inverse dose-response association between leisure-time PA and all-cause and cause-specific mortality in adults with major chronic diseases. Also, a more reduced risk of CVD-specific mortality was found among those with the same leisure-time PA doses than the reduced risk of all-cause and cancer-specific mortality. These findings suggest the survival benefits of leisure-time PA regardless of doses in adults with chronic diseases, particularly for those with CVD. Although limited previous studies focused on one specific chronic disease such as CVD (15), hypertension (24), and breast cancer (25) showed a similar dose-response relationship between PA and the risk of mortality, we found that the dose-response association was not only confined to those with specific chronic diseases (i.e., hypertension, heart disease, stroke, diabetes, or cancer) but also persisted among those with multiple chronic diseases (2 or more than 3).

PA has been regarded as a cost-effective treatment for most chronic diseases in clinical practice due to its established association with better health (26). Our findings showed that even a low level of leisure-time PA time (10–59 min/week) could result in substantial survival benefits in adults with major chronic diseases. Our findings also support that sufficient leisure-time PA time (i.e., more than 150 min of moderate-intensity PA, or 75 min of vigorous-intensity PA per week, or an equivalent combination) recommended by the 2020 PA Guidelines and World Health Organization PA guidelines (5) could result in additional health benefits for adults with chronic diseases (3). Despite the apparent benefits reported here and by others, data from the U.S. 2014 National Health Interview Survey showed that the proportion meeting sufficient total leisure-time PA levels in healthy adults was only 53.6%, and the proportion in those with chronic diseases was even lower (ranging from 26.1 to 48.6% depending on different specific diseases) (27). In this study, only 34.9% of adults with chronic diseases met the recommendation, suggesting effective measures are needed to enhance the leisure-time PA level among those with chronic diseases.

One important obstacle for adults with chronic diseases to meet the PA Guidelines is lack of time. For those with little time to perform sufficient PA, a low level of PA (e.g., 10 min per day or 60 min per week) should be prioritized, and levels increased according to willingness and capability. The other obstacle impeding adults to perform sufficient PA is the diagnosis of chronic diseases. Adults with chronic diseases such as CVD usually achieve a lower level of PA as they are limited by their chronic condition or are typically older with multiple co-morbidities (15). Our study has also shown that as the number of chronic diseases increases, those achieved less total leisure-time PA levels, which might be affected by the decline of physical functioning (28).

In addition, we found that both low and high levels of light-to-moderate and vigorous-intensity PA time could reduce the risk of mortality. Although vigorous leisure-time PA tended to be slightly better than light-to-moderate-intensity PA at some levels, especially for reduced risk of CVD-specific mortality for leisure-time PA ≥ 600 min/week, the reduced risks of mortality of both intensities were largely comparable. When adults with chronic diseases are unable to meet the minimum amount of PA recommended by the 2018 PA Guidelines, especially for those with multiple chronic diseases, the low level of the light-to-moderate intensity of PA time (which is more achievable or desired) according to their severity of chronic disease and abilities could be prioritized and recommended (3).

### Strengths and limitations

The major strength of this study is the large, nationally representative sample of the general population of U.S. adults with chronic diseases that used consistent methodology to collect information on demographics, lifestyle behaviors, and chronic diseases (29). Second, our two sensitivity analyses (stratification by type or number of chronic diseases; exclusion of participants who died within 2 years of baseline measurement) recovered similar results to our main findings. Our study also has limitations. First, recall bias might exist due to the self-report of leisure-time PA and other covariates. However, self-reports might result in regression dilution bias and thus shift the “true” association between leisure-time PA and mortality toward the null (30). Second, data on leisure-time PA was only obtained at baseline. We were, therefore, unable to consider the impact of changes in leisure-time PA levels during follow-up. Future studies with repeated measures of leisure-time PA are needed to evaluate its effect on mortality risk. Third, only leisure-time PA was used in this study as information on other types of PA such as occupation, household, and transportation PA were not collected (31). Thus, we were unable to determine the contribution of PA accumulated in different domains of PA on our outcomes. However, it has been shown that leisure-time PA exerted more significant protective effects on all-cause and cancer-specific mortality than transportation and household PA (31). Future studies are needed to further examine the association between domains of PA and all-cause and cause-specific mortality in chronic adults. Fourth, although many confounding factors were adjusted for, residual, and unmeasured confounding may have influenced our results. Fifth, information on the severity of chronic diseases was not available. Therefore, our findings that the highest level of leisure-time PA associated with decreased mortality should be generalized with caution, as participants in this group were more likely to have light-to-mild disease and less chronic diseases and severity of disease. Sixth, we focused on major chronic diseases (e.g., hypertension, heart disease, stroke, and cancer) without consideration of some other unavailable chronic diseases such as chronic kidney disease. Further studies on this issue among adults with more chronic diseases are needed to confirm our results. Seventh, the prognosis of cancer varied by the type of cancer, therefore, the findings on all cancer-specific mortality in the present study should be interpreted with caution. Eighth, muscle-strengthening activity might partly explain a higher BMI among participants with leisure-time PA ≥ 150 min/week compared with those with < 150 min/week, which was only used as a covariant in this study. Future studies are needed to assess the association of combined leisure-time PA and muscle-strengthening activity with the reduction of mortality risk. Ninth, the NHIS questionnaire measures leisure-time PA in bouts of 10 min or more per week. Therefore, despite we observed the dose-response association, where there were benefits of leisure-time PA which might come from bouts lasting longer than 10 min/week but not from shorter bouts, future studies are needed for further validation. Tenth, no dietary/nutrition information was assessed. Further studies are needed to examine the modified effects of dietary habits on the association between leisure-time PA and mortality risk.

## Conclusion

Low and high levels of leisure-time PA were associated with a reduction in risk of all-cause, CVD-, and cancer-related mortality among those with chronic disease compared with no leisure-time PA. There was an inversely non-linear association between leisure-time PA dose and risk of all-cause, CVD- and cancer-specific mortality. Light-to-moderate-intensity PA is largely comparable to vigorous PA time for reduced risk of all-cause and cancer-specific mortality, except for more reduced risks of CVD-specific mortality for vigorous PA ≥ 600 min/week. According to their abilities, adults with chronic disease should be encouraged to engage in leisure-time PA at least at a low dose, while the dose increases according to willingness and capability.

## Data availability statement

The datasets presented in this study can be found in online repositories. The names of the repository/repositories and accession number(s) can be found below: http://www.cdc.gov/nchs/nhis.htm.

## Ethics statement

The NHIS data are de-identified and do not include any protected health information, and the available data are public and exempt under the ethical review board of the corresponding author institution. The patients/participants provided their written informed consent to participate in this study.

## Author contributions

BX and MZ designed the study as the principal investigator. JS drafted the manuscript. HW and BX conducted the data analysis. CM, BX, MZ, and JS made critical revisions to the manuscript for important intellectual content. BX was the guarantor and attested that all the listed authors met the authorship criteria and that no others meeting the criteria had been omitted. All authors approved the final version of the manuscript.

## Footnotes

1
http://www.cdc.gov/nchs/nhis.htm
