# Supplementary material for: Dose-response association of leisure time physical activity with mortality in adults with major chronic diseases
Source: Front Nutr. 2022 Dec 21;9:1048238. doi: 10.3389/fnut.2022.1048238 (PMC9811256; doi:10.3389/fnut.2022.1048238)
Supplement: Supplementary file 1 [file Table_1.DOC]

| **Table S1. Association between total leisure-time physical activity level and all-cause mortality stratified by age, sex and race/ethnicity** | | | | | | | | |
| --- | --- | --- | --- | --- | --- | --- | --- | --- |
|  | | | | | | | | |
| Group | Leisure-time physical activity level, minutes/week | | | | | | | |
|  | 0 | 10-59 | 60-149 | 150-299 | 300-449 | 450-799 | 800-1499 | ≥1500 |
| Age, yrs |  |  |  |  |  |  |  |  |
| 30-49 | 1.00 | 0.78 (0.63-0.96) | 0.71 (0.61-0.82) | 0.63 (0.55-0.74) | 0.56 (0.47-0.66) | 0.64 (0.54-0.76) | 0.48 (0.38-0.60) | 0.57 (0.44-0.74) |
| 50-69 | 1.00 | 0.78 (0.71-0.87) | 0.72 (0.67-0.76) | 0.60 (0.56-0.64) | 0.58 (0.53-0.64) | 0.55 (0.50-0.61) | 0.53 (0.48-0.60) | 0.57 (0.49-0.67) |
| 70-84 | 1.00 | 0.75 (0.69-0.82) | 0.75 (0.72-0.79) | 0.65 (0.62-0.69) | 0.61 (0.56-0.65) | 0.55 (0.51-0.60) | 0.57 (0.51-0.62) | 0.52 (0.45-0.60) |
| Sex |  |  |  |  |  |  |  |  |
| Men | 1.00 | 0.77 (0.70-0.84) | 0.75 (0.71-0.80) | 0.64 (0.60-0.67) | 0.59 (0.55-0.63) | 0.55 (0.51-0.59) | 0.55 (0.50-0.60) | 0.55 (0.49-0.62) |
| Women | 1.00 | 0.76 (0.70-0.83) | 0.72 (0.68-0.75) | 0.62 (0.59-0.66) | 0.60 (0.55-0.65) | 0.57 (0.52-0.64) | 0.53 (0.47-0.59) | 0.56 (0.46-0.68) |
| Race/ethnicity |  |  |  |  |  |  |  |  |
| White | 1.00 | 0.74 (0.69-0.80) | 0.73 (0.70-0.77) | 0.62 (0.60-0.65) | 0.59 (0.56-0.63) | 0.55 (0.52-0.59) | 0.52 (0.48-0.57) | 0.54 (0.48-0.60) |
| Black | 1.00 | 0.78 (0.67-0.90) | 0.76 (0.69-0.85) | 0.68 (0.59-0.77) | 0.58 (0.50-0.67) | 0.61 (0.50-0.75) | 0.68 (0.55-0.84) | 0.61 (0.45-0.83) |
| Hispanic | 1.00 | 1.07 (0.85-1.35) | 0.77 (0.66-0.89) | 0.64 (0.53-0.76) | 0.66 (0.54-0.81) | 0.62 (0.48-0.80) | 0.60 (0.47-0.76) | 0.68 (0.45-1.03) |
| Other | 1.00 | 0.85 (0.56-1.31) | 0.62 (0.47-0.82) | 0.69 (0.53-0.89) | 0.63 (0.46-0.86) | 0.68 (0.44-1.04) | 0.69 (0.45-1.07) | 0.41 (0.20-0.83) |
| Data are presented as hazard ratios (95% confidence intervals) adjusted for sex, age, race/ethnicity, education, marital status, body mass index, smoking, alcohol intake, number of chronic diseases, and muscle-strengthening activity | | | | | | | | |

| **Table S2. Association between total leisure-time physical activity level and all-cause mortality stratified by chronic disease type and number at baseline** | | | | | | | | |
| --- | --- | --- | --- | --- | --- | --- | --- | --- |
|  | | | | | | | | |
| Chronic disease at baseline | Leisure-time physical activity level, minutes/week | | | | | | | |
|  | 0 | 10-59 | 60-149 | 150-299 | 300-449 | 450-799 | 800-1499 | ≥1500 |
| Hypertension (n=127488) | 1.00 | 0.78 (0.72-0.83) | 0.73 (0.70-0.76) | 0.63 (0.60-0.66) | 0.59 (0.56-0.63) | 0.58 (0.54-0.62) | 0.55 (0.50-0.59) | 0.56 (0.50-0.63) |
| Heart disease (n= 52700) | 1.00 | 0.73 (0.67-0.80) | 0.72 (0.68-0.77) | 0.63 (0.60-0.68) | 0.59 (0.54-0.64) | 0.53 (0.48-0.58) | 0.51 (0.46-0.57) | 0.47 (0.40-0.55) |
| Stroke (n=12359) | 1.00 | 0.83 (0.71-0.97) | 0.69 (0.62-0.77) | 0.66 (0.58-0.76) | 0.59 (0.50-0.69) | 0.59 (0.48-0.73) | 0.57 (0.47-0.69) | 0.48 (0.35-0.66) |
| Diabetes (n=38069) | 1.00 | 0.77 (0.69-0.86) | 0.74 (0.68-0.80) | 0.62 (0.57-0.68) | 0.54 (0.49-0.61) | 0.54 (0.48-0.61) | 0.55 (0.47-0.64) | 0.64 (0.51-0.81) |
| Cancer (n=34393) | 1.00 | 0.78 (0.69-0.87) | 0.70 (0.65-0.75) | 0.60 (0.55-0.65) | 0.53 (0.48-0.59) | 0.52 (0.47-0.58) | 0.47 (0.41-0.53) | 0.47 (0.40-0.56) |
| 1 chronic disease (n=102416) | 1.00 | 0.78 (0.71-0.86) | 0.77 (0.73-0.82) | 0.64 (0.61-0.68) | 0.63 (0.58-0.68) | 0.59 (0.54-0.64) | 0.59 (0.53-0.65) | 0.58 (0.51-0.67) |
| 2 chronic diseases (n=46717) | 1.00 | 0.75 (0.67-0.83) | 0.72 (0.68-0.77) | 0.64 (0.59-0.69) | 0.61 (0.56-0.67) | 0.58 (0.52-0.64) | 0.55 (0.49-0.62) | 0.60 (0.50-0.72) |
| ≥3 chronic diseases (n=21446) | 1.00 | 0.79 (0.70-0.88) | 0.74 (0.68-0.80) | 0.64 (0.58-0.71) | 0.55 (0.48-0.62) | 0.55 (0.48-0.64) | 0.50 (0.42-0.60) | 0.48 (0.36-0.62) |
| Data are presented as hazard ratios (95% confidence intervals) adjusted for sex, age, race/ethnicity, education, marital status, body mass index, smoking, alcohol intake, and muscle-strengthening activity | | | | | | | | |

| **Table S3. Association between total leisure-time physical activity level and all-cause and cause-specific mortality after exclusion of patients who died within the first 2 years** | | | | | | | | |
| --- | --- | --- | --- | --- | --- | --- | --- | --- |
|  | | | | | | | | |
| Cause of deaths | Leisure-time physical activity level, minutes/week | | | | | | | |
|  | 0 | 10-59 | 60-149 | 150-299 | 300-449 | 450-799 | 800-1499 | ≥1500 |
| All-cause | 1.00 | 0.82 (0.77-0.87) | 0.77 (0.74-0.80) | 0.67 (0.64-0.70) | 0.65 (0.62-0.69) | 0.61 (0.57-0.65) | 0.59 (0.55-0.63) | 0.60 (0.54-0.67) |
| Cardiovascular disease | 1.00 | 0.74 (0.64-0.85) | 0.74 (0.68-0.80) | 0.59 (0.53-0.65) | 0.59 (0.52-0.66) | 0.55 (0.48-0.63) | 0.51 (0.44-0.60) | 0.40 (0.31-0.51) |
| Cancer | 1.00 | 0.88 (0.77-1.00) | 0.86 (0.79-0.94) | 0.73 (0.67-0.80) | 0.72 (0.64-0.81) | 0.74 (0.66-0.84) | 0.62 (0.53-0.72) | 0.78 (0.64-0.95) |
| Data are presented as hazard ratios (95% confidence intervals) adjusted for sex, age, race/ethnicity, education, marital status, body mass index, smoking, alcohol intake and, number of chronic diseases, and muscle-strengthening activity | | | | | | | | |
